# Land Cover and Seasonal Variations Shape Soil Microbial Communities and Nutrient Cycling in Madagascar Tropical Forests

**DOI:** 10.1007/s00248-025-02561-w

**Published:** 2025-06-04

**Authors:** Vahatra Rakotonindrina, Andry Andriamananjara, Tantely Razafimbelo, Takanori Okamoto, Papa Saliou Sarr

**Affiliations:** 1 Laboratoire Des Radioisotopes, University of Antananarivo, Route d’Andraisoro, BP 3383, 101 Antananarivo, Madagascar; 2 Crop, Livestock and Environment Division, Japan International Research Center for Agricultural Sciences, 1-1 Ohwashi, Tsukuba, Ibaraki 305-8686 Japan

**Keywords:** Land cover, Microbial community, Phosphorus cycling, PH, Madagascar

## Abstract

Understanding how land cover and seasonal variations influence soil microbial communities and nutrient cycling is crucial for sustainable land management in tropical forests. However, such investigations are limited in Madagascar’s tropical ecosystems. This study investigated the impacts of land cover types and seasonal variations on soil properties and microbial communities in the tropical forest region of Andasibe, Madagascar. Soil samples were collected from four land cover types—tree fallow (TSA), shrub fallow (SSA), eucalyptus forest (EUC), and degraded land (TM)—across three seasonal periods: the dry season, the start of the rainy season, and the end of the rainy season. Both land cover and sampling season affected soil pH and available P, whereas total nitrogen, soil organic carbon, and the C/N ratio were affected only by land cover. The soil organic carbon and total nitrogen concentrations were greater in TM. NextSeq sequencing of the *16S rRNA* gene and *ITS* regions of the nuclear rRNA operon revealed distinct microbial community compositions across land covers, with greater diversity in the TSA and SSA. Bacteria are more sensitive to seasonal changes than are fungi, with phosphate-solubilizing (*gcd*) and phosphate-mineralizing (*phoD*) genes being more abundant in the rainy season, emphasizing the role of microbes in nutrient availability under different climatic conditions. Principal component analysis highlighted SSA as a hotspot for microbial activity, reinforcing the potential of shrub ecosystems in soil restoration. These findings reveal strong land cover and seasonal effects on soil microbial functions, with implications for nutrient cycling, ecosystem resilience, and sustainable land management in tropical forest landscapes.

**Supplementary Information:**

The online version contains supplementary material available at 10.1007/s00248-025-02561-w.

## Introduction

Soil microorganisms play a crucial role in ecosystem functioning by facilitating processes such as organic matter decomposition, nutrient cycling, and plant growth [1]. Microbial biomass in soils accounts for 0.6–1.1% of soil organic carbon [2], and microorganisms promote plant growth by providing essential nutrients, including phosphorus (P), through solubilization and mineralization. P is vital for alleviating plant abiotic stress, supporting root formation, increasing nutrient uptake, and regulating photosynthesis and stomatal function [3]. In P-limited soil, particularly in highly weathered tropical environments where bioavailable P is minimal [4], competition for P between soil microorganisms and plants can hinder plant growth, resulting in crop yield losses of up to 40% [5]. To survive in such environments, microorganisms must balance carbon (C):nitrogen (N):P ratios to survive, with element acquisition and partitioning being key factors [6]. Understanding how nutrient competition between microbes and plants, especially for phosphorus, varies under different environmental conditions—such as in tropical forests or disturbed lands—is essential for developing better strategies for land management and ecosystem restoration.

Soil texture and factors such as pH, moisture, temperature, and plant diversity significantly influence microbial communities [7–9]. Additionally, root exudates, which act as nutrient sources, selectively promote specific microorganisms in the rhizosphere, and environmental stress can alter the quantity and composition of these exudates [10]. Microbial interactions, particularly nutrient competition, also play crucial roles in shaping community structure and defining ecological niches [11].

The dynamics of microbial communities occur over multiple timescales: short-term changes from disturbances, seasonal shifts driven by climate and plant activity, long-term changes linked to vegetation shifts, and the longest scale related to soil formation [12]. In tropical and subtropical regions, precipitation is a major factor that shapes microbial communities [13]. However, land cover dynamics, such as deforestation and slash-and-burn agriculture, significantly alter microbial communities [14]. In Madagascar, diverse ecosystems are under pressure from human activities and climate change, which impact soil properties and biological functions. While natural ecosystem recovery can be slow, reforestation efforts have the potential to accelerate process, provided certain guidelines are followed to ensure their success [15]. For example, post-fire planting has been shown to enhance forest recovery in burned areas, increasing regrowth rates by 25% within five to eight years after planting [16]. However, there is limited research on how soil microbial communities contribute to the recovery of ecosystem functions, such as nutrient cycling and soil fertility, following disturbances. In-depth investigations of microbial community changes during reforestation could provide valuable insights into the role of microbes in ecosystem recovery. Furthermore, it is crucial to examine how seasonal variations, such as changes in temperature, precipitation, and plant activity, affect microbial communities in tropical forests, particularly in Madagascar. Longitudinal studies that assess microbial changes across seasons could help understand how microbial communities dynamically respond to climatic fluctuations. While much research has focused on carbon storage [17] and biogeochemical processes [18], little research has been conducted to assess the specific impact of land use changes on the biological properties of Madagascar soil. Therefore, it is necessary to investigate the specific effects of land use changes on microbial community composition, structure, and function. This can be achieved by comparing microbial diversity, functional genes (e.g., those related to P solubilization and mineralization, nitrogen fixation), and nutrient cycling across land covers.

This study examined the dynamics of soil bacterial and fungal communities, functional genes related to P mineralization and solubilization, nitrogen fixation, and soil properties across various land covers and seasons in Madagascar. We hypothesize that microbial structure is influenced by land cover type and seasonal variations, which reflect the impact of environmental factors on ecosystem function. An integrated approach combining genomic, soil chemistry, and ecosystem data could reveal how microbial communities mediate nutrient cycling processes, particularly P and N, in different land cover types. This could further enhance our understanding of the broader ecological implications of land use changes for soil fertility and ecosystem health.

## Materials and Methods

### Study Area

The study was conducted in the tropical forest region of Andasibe (Supplementary Fig. S1A), eastern Madagascar (18°92′S, 48°41′E), which has a hot, humid climate with an annual rainfall of 2500 mm and temperatures ranging from 18 °C to 24 °C [17]. We obtained empirical data from local weather stations for the years 2021, 2022, and 2023. From these datasets, we extracted data covering the sampling period (May 2022–May 2023). For each month, daily mean values were calculated by dividing the total monthly values by the number of days in that month. Precipitation was reported both as average daily and total monthly amounts. Since all sampling sites were located within the area covered by the same local weather station, site-specific meteorological values were not available. As a result, the recorded values were uniform across sites but varied throughout the different sampling periods (Supplementary Table S1). Among the sampled months, June was the driest, with an average daily precipitation of 5.42 mm and a total monthly precipitation of 162.59 mm, although the driest period of the year is typically from August to September. In contrast, precipitation totaled 323.54 mm in December 2022 and 477.49 mm in March 2023. Relative humidity was slightly higher in June (92.94%) than in December and March (88.27% and 90.89%, respectively), which may be attributed to the lower maximum temperatures in June (Tmax = 18.11 °C) compared to December (Tmax = 24.72 °C) and March (24.62 °C). Soil samples, predominantly ferralsols, according to the FAO classification system, were collected from four land cover types: tree fallow (TSA), shrub fallow (SSA), three-decade-old eucalyptus forest (EUC), and degraded land (TM). Land cover types were classified based on indicator species [19], as shown in Supplementary Table S2. TSA was dominated by native tree species such as *Trema orientalis* and *Harungana madagascariensis*. In SSA, the indicators species included *Psidisa altissima*, *Rubus moluccanus*, and *Lantana camara*. The EUC site was dominated by Eucalyptus species, while TM was characterized by the presence of *Imperata cylindrica*. The sampling sites represented a chronosequence of ecosystem restoration, enabling the examination of microbial dynamics in response to land cover changes and seasonal variations.

### Vegetation Diversity Survey

In May 2023, following the rainy season, an ecological survey was conducted to assess plant species richness and abundance using Shannon’s diversity index. This month was deliberately chosen to capture peak plant diversity after the rainy season. In tropical ecosystems, the end of the rainy season typically corresponds with maximum vegetation growth and floral development, as many plant species complete key phenological stages such as germination, flowering, and leaf flushing during this period. Consequently, plant diversity, both in terms of both richness and detectability, is often highest during this transitional period. This timing aligns with standard practices tropical biodiversity research, where surveys are commonly conducted at the end of the wet season for similar ecological practical reasons [20]. A 20-m radius around each sampling point was surveyed according to the forest inventory protocol [17], and the diversity index (H’) was calculated as follows:

$$H^{\prime}=-{\sum }_{i=1}^{S}pi \mathrm{ln}(pi)$$

where:

*H’*: Shannon diversity index.
*S*: total number of species.
*pi*: proportion of individuals belonging to the *i*th species.

Plant species evenness was assessed via Pielou’s evenness index (E):

$$E=\frac{{H}^{\prime}}{\mathrm{ln}(S)}$$

where:

*H’*: Shannon diversity index.
*S*: total number of species.

### Soil Sampling

A total of 48 soil samples (12 per plot) were collected at three seasonal intervals: the dry season (June 2022, hereafter referred to as DS), the start of the rainy season (December 2022, hereafter referred to as SRS), and the end of the rainy season (March 2023, hereafter referred to as ERS). These intervals allowed us to examine seasonal variations in microbial communities. The soil was sampled at a depth of 0–10 cm, with four replicates per time point. In the field, samples were sieved through a 2-mm mesh to remove plant roots and coarse particles and then separated into two portions: one for chemical analysis and one for microbiological analysis. The microbiological samples were kept on ice until they reached the laboratory and then stored at − 20 °C until DA extraction, while the chemical samples were air-dried, ground and sieved through 0.2 mm mesh.

### Soil Analysis

#### Soil Physicochemical Analysis

The soil texture, organic carbon (SOC), total nitrogen (N_tot_), total phosphorus (P_tot_), available phosphorus (P Olsen), pH, and C/N ratio were analyzed. The soil texture was determined via granulometric fractionation via the pipette method [17]. SOC was measured via the Walkley‒Black method [21], N_tot_ via the Kjeldahl method [22] and P_tot_ via the blue molybdenum colorimetric method. Available P was assessed via the Olsen method [23], and the pH was measured in a 1:25 soil-to-water solution.

#### Soil DNA Extraction

DNA was extracted from 0.4 g of frozen soil via the FastDNA SPIN Kit for Soil (MP Biomedicals, USA) according to the manufacturer’s protocol. The DNA was then purified via the gDNA Clean-Up System (Qiagen) and quantified with a Qubit dsDNA HS Assay Kit (Thermo Fisher Scientific, USA). The purified DNA was sent to the Japan International Research Center for Agricultural Sciences (JIRCAS) for further analysis, where microbial gene abundance was determined via real-time PCR, and microbial composition was analyzed via Illumina NextSeq 1000 sequencing.

#### Microbial Gene Abundance by qPCR

Real-time PCR was used to quantify microbial genes, including total bacteria (*16S rRNA* gene), total fungi (*ITS* gene), glucose dehydrogenase (*gcd* gene), alkaline phosphatase (*phoD* gene), betaine, and nitrogen fixation (*nifD* gene) genes. The soil DNA was diluted 50-fold to minimize inhibitors (amplifications unsuccessful with the standard tenfold dilution), and PCR protocols for *16S rRNA* and *ITS rRNA* [24] with primers sourced from Bergkemper et al. [25] for *phoD*, Glass and Donaldson [26] for betaine, and Stoltzfus et al. [27] for *nifD* were used. The *gcd* and *phoD* genes are microbial markers for phosphate solubilization and mineralization, respectively. The annealing temperatures for the *nifD* and betaine genes were 52 °C and 55 °C, respectively. The amplification efficiencies and r^2^ values are provided in Supplementary Table S3.

#### Illumina NextSeq 1000 Sequencing of Bacteria and Fungi

DNA from the soil samples collected in DS and SRS was sequenced at Bioengineering Lab. Co. (Kanagawa, Japan). The V4 region of the *16S rRNA* gene and the *ITS2* region of the nuclear rRNA operon were amplified via the primer sets 515 F-modified [28] and 806R-modified [29] gene and the primer sets 1 st-gITS_MIX2/1 st -ITS4_MIX2 [30], respectively. The first PCR mixture of the 16S *rRNA* gene and *ITS* gene consisted of a 10-µL reaction mixture consisting of 5 µL of 2 × PCR Buffer for KOD FX Neo, 2 µL of dNTPs (each 2 mM), 0.2 µL of each primer at 10 µM, 1 µL of template DNA, 0.2 µl of KOD FX neo (1.0 U µL^−1^) from TOYOBO Co., Ltd. (Kyoto, Japan), and 1.4 µl of nuclease-free water. The thermal cycling conditions for the first PCR were as follows: 94 °C for 2 min; 30 cycles of 98 °C for 10 s, 50 °C for 30 s, and 68 °C for 30 s; 68 °C for 7 min for *16S rRNA* and 94 °C for 3 min; 35 cycles of 94 °C for 15 s, 50 °C for 30 s, and 68 °C for 1 min; and 68 °C for 7 min for *ITS*. The PCR products were cleaned with VAHTS DNA Clean Beads (Nanjing Vazyme Biotech Co., Ltd., Nanjing, China). A second PCR was performed following the manufacturer’s instructions and sequenced on an Illumina NextSeq 1000 system (Illumina Inc., San Diego, CA, USA). The raw data were defined as raw tags after removing the barcode and PCR primer sequences via the fastq_barcode_splitter tool from FASTX-Toolkit (ver. 0.0.14). The sequences with a quality value of less than 20 were removed via sickle (ver. 1.33) [31]. The paired-end read-merging script FLASH (ver. 1.2.11) [32] was used to merge the reads with a minimum overlap of 10 bp. After removing chimeric and noise sequences, amplicon sequence variants (ASVs) were generated via the dada2 plugin in QIIME2 (ver. 2024.10) [33]. ASVs were assigned taxonomy via the feature-classifier plugin in QIIME2 against the Greengenes database (ver. 13_8) [31] for 16S rRNA and the UNITE database (ver. 10.0) [34] for ITSs. For the 16S rRNA amplicon data, sequences classified as mitochondrial or chloroplast DNA were also removed. The remaining ASVs were processed via the R package vegan 2.6–6.1 to calculate alpha diversity metrics, including the Shannon diversity and Simpson indices for the *16S rRNA* and *ITS* genes. Beta diversity was analyzed via nonmetric multidimensional scaling (NMDS) on the basis of Bray–Curtis dissimilarity. NMDS plots were generated from the weighted UniFrac base matrix in the R package vegan, following Sasaki et al. [35]. The microbial community composition at the phylum, class, and family levels was determined for each land cover type. Sequence information has been deposited in DDBJ/ENA/GenBank under the BioProject accession number PRJDB19896 and DDBJ. Sequence Read Archive accession numbers DRR627433–DRR627464 for the 16S rRNA sequences and DRR627465–DRR627496 for the ITS sequences.

### Statistical Analysis

Statistical analyses were performed via R (version 2024.4.2.764) [36]. Two-way ANOVA was used to test differences in microbial abundance and soil properties across land cover types and seasonal intervals via the ‘lm’ function from the ‘car’ package. Data normality and variance homogeneity were verified via the Shapiro‒Wilk test and Bartlett test, respectively. Significant differences were further analyzed with the Student‒Newman‒Keuls post hoc test at *p* < 0.05. PERMANOVA was applied for nonnormally distributed variables.

Pearson’s correlation tests (*p* < 0.05) were used to evaluate the relationships between the soil and climate parameters. Multivariate regression was used to assess the effects of soil variables on microbial abundance. Collinear variables were excluded from the initial model via the “vif” function, retaining only those with variance inflation factors (VIFs) less than 5. Model optimization was achieved through stepwise backward and forward regression on the basis of the Akaike information criterion (AIC), with less significant variables excluded iteratively. The relative importance of the final predictors was calculated via the “calc.relimp” function from the relaimpo package. Principal component analysis (PCA) was performed to explore the relationships between soil microbial and physicochemical variables, highlighting the potential effects of soil properties on microbial dynamics. We first performed standard assumption checks prior to the analysis to assess the suitability of the data for PCA. The Kaiser–Meyer–Olkin (KMO) measure of sampling adequacy was 0.5, which is considered the minimum acceptable threshold, particularly in studies with small sample sizes [37]. Bartlett’s test of sphericity was 3.181425e-182, which is highly significant (*p* < 0.001), indicating that the variables were sufficiently correlated to justify the use of PCA.

## Results

### Vegetation Community Composition

TSA had the highest plant Shannon diversity index, followed by SSA, whereas TM had the lowest Shannon and Pielou’s evenness indices, indicating dominance by a few species (Table 1). Floristic analysis revealed that, compared with the other land covers, SSA and TSA supported more diverse plant communities (Supplementary Fig. S1B).

**Table 1:** Shannon’s diversity and Pielou’s evenness indices of land cover types

Site	Shannon diversity index (H’)	Pielou evenness index (E)
TSA	2.99	0.41
SSA	2.61	0.34
EUC	2.40	0.33
TM	0.96	0.10

TSA: Tree fallow, SSA: Shrub fallow, EUC: Three-decade-old Eucalyptus forest, TM: degraded land

### Soil Physicochemical Properties

Distinct soil texture groups were observed across the four land covers (Supplementary Fig. 2), with clay contents ranging from 27% to 49.5%, sand contents ranging from 33.23% to 60.36%, and silt contents ranging from 12.7% to 21.23%. EUC had clay soil, SSA had sandy-clay and sandy-clay-loam, and TM and TSA, which are located 300-m apart, shared textures between sandy-clay/clay and clay-loam/clay. Soil chemical properties, including pH, total N, total C, the C/N ratio, and available P, were significantly affected by both land cover and season. The total P (*p* < 0.05), available P (*p* < 0.001), and pH (*p* < 0.001) varied seasonally, with significant interactions between land cover and season for pH and total P (Table 2). Seasonal dynamics played a key role, with available P being most sensitive to land cover in DS and the C/N ratio being more influenced by land cover in SRS and ERS.

**Table 2:** Land cover type and season effects on soil chemical properties (ANOVA)

	P_Ols_	Ptot	pH	Corg	Ntot	C/N
All data						
Land cover (A)	***	n.s	**	**	***	**
Season (B)	***	*	***			n.s
(A)*(B)	n.s	*	**	n.s	n.s	n.s
% variance explained (R^2^)	0.82***	0.42*	0.86***	0.39*	0.63***	0.34
Dry season						
Land cover (A)	***	n.s	**	n.s	**	n.s
% variance explained (R^2^)	0.77***	0.06	0.66**	0.39	0.71**	0.39
Start rainy season						
Land cover (A)	*	n.s	n.s	n.s	**	*
% variance explained (R^2^)	0.6*	0.25	0.29	0.34	0.66**	0.56*
End rainy season						
Land cover (A)	**	**	***	n.s		***
% variance explained (R^2^)	0.67**	0.65**	0.75***	0.2	0.42	0.76***

Significance level: n.s: not significant, * < 0.05, ** < 0.01, *** < 0.001

P_ols_: Olsen P, P_tot_: total phosphorus, C_org_, organic carbon, N_tot_: total nitrogen

**Fig. 1:**
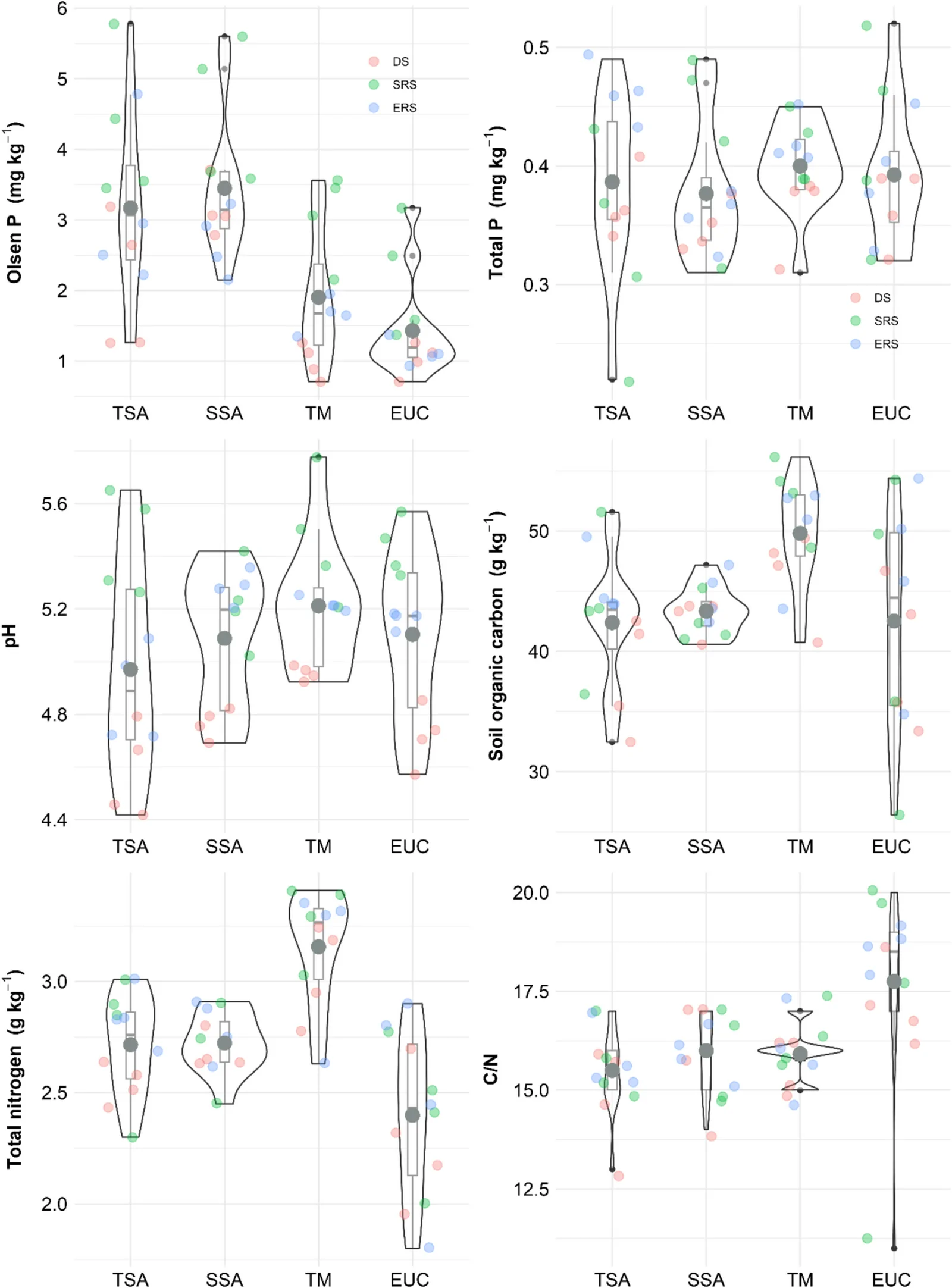
Chemical properties. TSA: Tree fallow, SSA: Shrub fallow, TM: Degraded land, EUC: Eucalyptus forest plantation, DS: dry season, SRS: start of rainy season, ERS: end of rainy season, P: Phosphorus

The chemical properties varied by season, with P_Ols_ and pH being greater in SRS (Fig. 1), and were influenced by land cover (Table 2). The SSA and TSA had the highest mean P_Ols_, whereas the EUC and TM had the lowest. The total P slightly increased in SSA and TM during SRS. The soil pH was lowest in DS and highest in SRS. The soil organic carbon content was greater in TM, the TN content was greater in TM, and the C/N ratio was highest in EUC.

### Relative Abundance of Microbial Genes

**Fig. 2:**
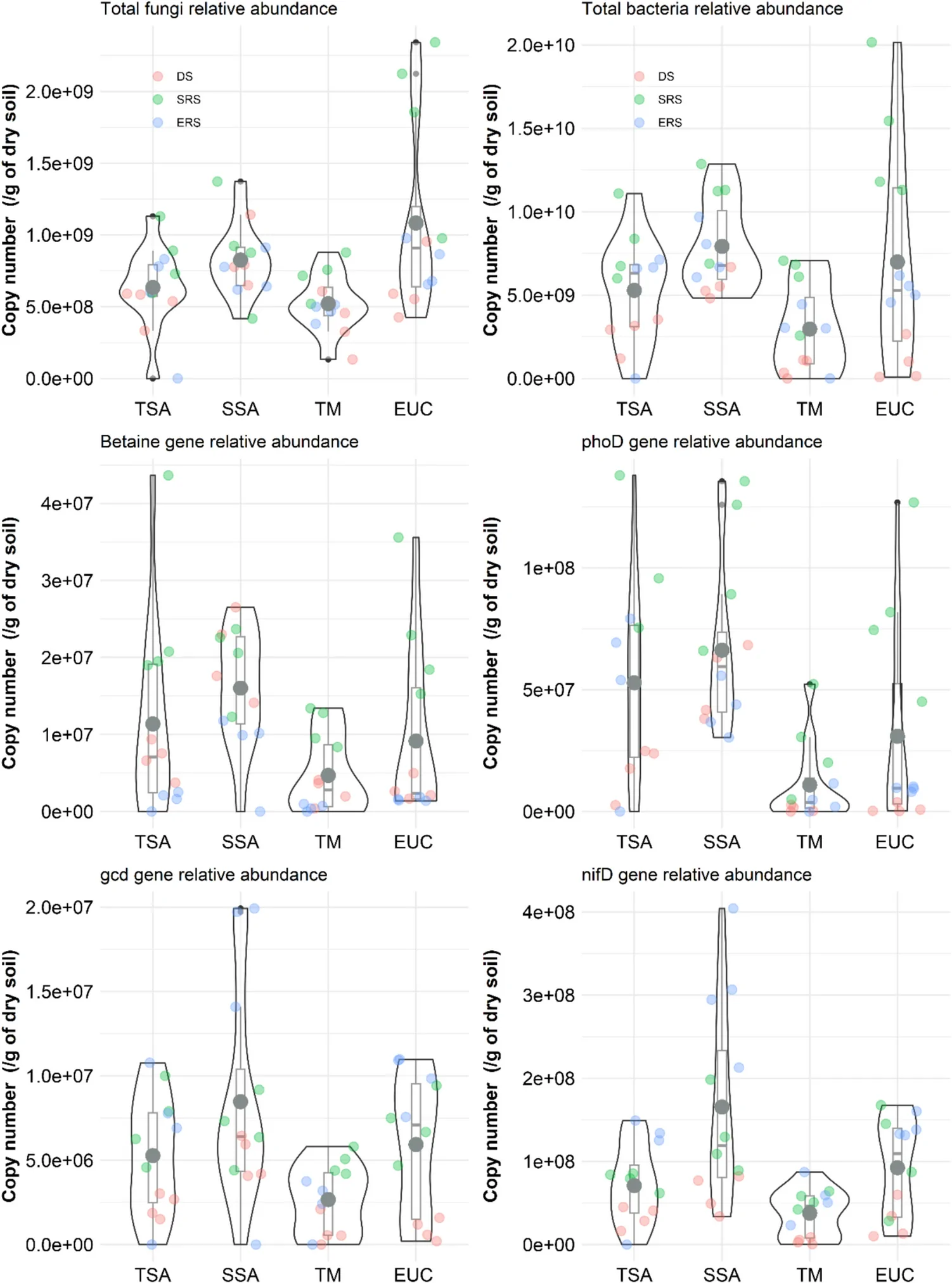
Microbial relative abundance according to land cover types. TSA: Tree fallow, SSA: Shrub fallow, TM: Degraded land, EUC: Eucalyptus forest plantation, DS: dry season, SRS: start of rainy season, ERS: end of rainy season

Both land cover and season significantly influenced the abundance of total bacteria, fungi, and functional genes (*gcd*, *phoD*, betaine, and *nifD*) (Table 3). The bacterial abundance was more sensitive to seasonal changes than the fungal abundance was (*p* < 0.001), with the microbial abundance being lowest in DS and ERS and highest in SRS (Fig. 2). Most genes followed this trend, except for *gcd* and *nifD*, which were more abundant in ERS. The bacterial abundance peaked in SRS, whereas *gcd* and *nifD* were more abundant in ERS. SSA presented the highest *phoD* gene abundance, peaking in SRS, and the highest *nifD* gene abundance, peaking in ERS (Fig. 2). The SSA also had the highest overall microbial gene abundance, followed by the TSA and EUC. Fungal abundance was slightly greater in EUC, whereas betaine gene abundance was most influenced by land cover and season, with SSA showing the highest and TM the lowest levels.

**Table 3:** Significance of the effect of Land cover type and seasonal variability on soil microbial relative abundance

	*16S rRNA*	*ITS*	*gcd*	*phoD*	betaine	*nifD*
**All data**						
Land cover (A)	***	***	***	***	***	***
Season (B)	***	***	***	***	***	***
(A)*(B)	***		**	*	***	n.s
% variance explained (R^2^)	0.86***	0.65***	0.79***	0.79***	0.89***	0.83***
**Dry season**						
Land cover (A)	*	*	**	***	***	**
% variance explained (R^2^)	0.54**	0.50**	0.72**	0.79**	0.87***	0.63**
**Start rainy season**						
Land cover (A)	***	**	n.s	*	*	
% variance explained (R^2^)	0.74**	0.64**	0.26	0.54*	0.56*	0.42
**End rainy season**						
Land cover (A)	***	*	**	***	***	***
% variance explained (R^2^)	0.79***	0.60	0.67*	0.82***	0.79**	0.82***

Significance level: n.s: not significant, * < 0.05, ** < 0.01, *** < 0.001

*16S rRNA*: bacterial gene, *ITS*: fungal gene, *gcd*: glucose dehydrogenase gene, *phoD*: alkaline phosphatase gene, *nifD*: nitrogen fixation gene

### *Soil Microbial Composition Determined *via* Illumina NextSeq*

The total raw reads for the *16S rRNA* and *ITS* genes, along with valid *16S rRNA* reads and ASVs, are shown in Supplementary Table S4. The *16S rRNA* gene generated 13,339 to 16,427 valid reads and 531 to 810 ASVs, whereas the *ITS* gene produced 42,591 to 44,166 reads and 240 to 428 ASVs. A significant interaction between the sampling period (P) and land cover (LC) affected the bacterial α diversity indices (Supplementary Table S5), with the Shannon index ranging from 5.567 to 5.974 and the Simpson index ranging from 0.9897 to 0.9952. Higher values were observed in DS than in SRS. In DS, TM had the highest Shannon index, whereas EUC had the lowest. In the SRS, the TSA had the highest Shannon and Simpson indices. β-Diversity analysis based on Bray‒Curtis dissimilarities revealed greater variation in TM and EUC than in the other land covers, whereas TSA and SSA resulted in similar bacterial community compositions (Supplementary Fig. S3).

Fungal diversity did not significantly differ between P and LC, and the period did not affect the α diversity indices (Supplementary Table S6). TSA presented the highest fungal diversity, whereas EUC presented the lowest fungal diversity. NMDS analysis revealed more homogeneous β diversity in TM and EUC, whereas TSA and SSA were similar. Seasonal variation did not significantly affect fungal β diversity (Supplementary Fig. S4).

**Fig. 3:**
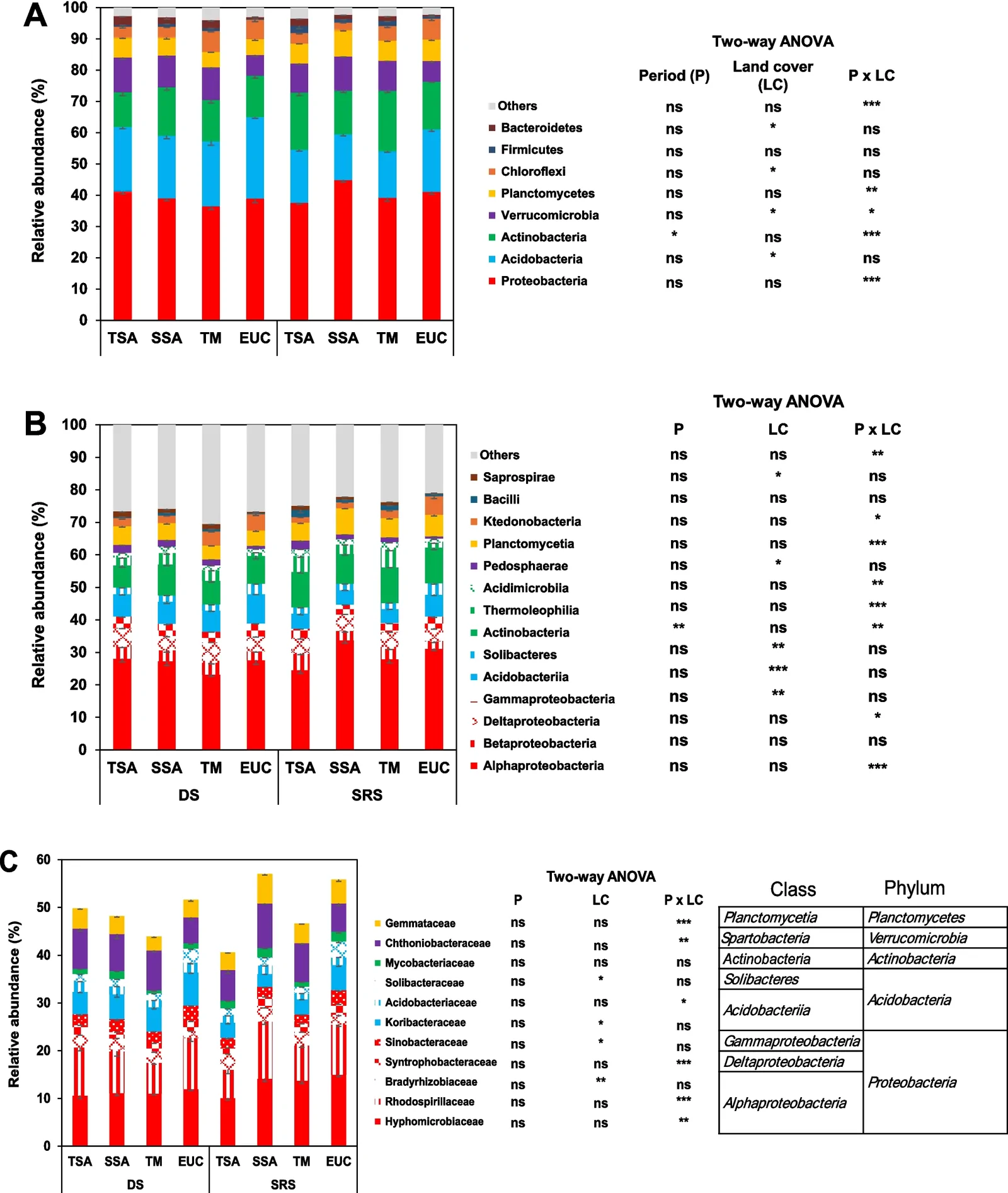
Comparisons of (**A**) phylum level, (**B**) class level (representative 8 bacterial phyla with % ASV > 1), and (**C**) family level (representative 10 families with %ASV > 2) of relative abundances of soil microbial 16S rRNA gene obtained from four land use types. TSA: Tree fallow, SSA: Shrub fallow, TM: Degraded land, EUC: Eucalyptus forest plantation, DS: dry season, SRS: start of rainy season (December). Data are means relative abundances − SE (*n* = 4). ***, **, and * indicate significance at *p* < 0.001, 0.01, and 0.05, respectively, ns: indicates not significant. *p*-values are based on the results of two-way ANOVA

*Proteobacteria* dominated the bacterial phyla, accounting for 36.51% to 44.80% of the total abundance (Fig. 3A), and were unaffected by season or land cover. *Acidobacteria* (14.67%–25.98%) were influenced by land cover, with a relatively high abundance in EUC. *Actinobacteria* (11.13%–19.28%) were more affected by the sampling period, peaking in SRS. A significant interaction between P and LC affected *Verrucomicrobiota*, which was more abundant in the TSS, SSA, and TM than in the EUC during DS but increased in the SSA in the SRS. *Chloroflexi* were more abundant in TM and EUC, whereas *Bacteroidetes* were more abundant in TSA, SSA, and TM across both periods. At the class level (Fig. 3B), *Alphaproteobacteria* dominated *Proteobacteria* (23.25% to 33.80%). LC influenced *Gammaproteobacteria* abundance, which was greater in EUC and lower in TM. *Acidobacteriia* and *Solibacteres* were more abundant in EUC and slightly less abundant in TM. The abundance of Actinobacteria was greater in the SRS than in the DS. *Pedosphaerae* and *Saprospirae* were more abundant in TSA, SSA, and TM than in EUC, with TSA showing higher abundance than SSA and TM in SRS.

Figure 3C presents the abundances of ten representative bacterial families whose ASV percentages were greater than 2%. A significant interaction effect between P and LC was observed for six families. Although P had no effect, LC influenced the abundance of some families. *Bradyrhizobiaceae* (*Alphaproteobacteria*) was most abundant in TSA and SSA and was less abundant in EUC. *Sonoacteraceae* (*Gammaproteobacteria*), *Koribacteraceae* (*Acidobacteria*), and *Solibacteriaceae* (*Solibacteres* class of Actinobacteria) were more abundant in EUC.

**Fig. 4:**
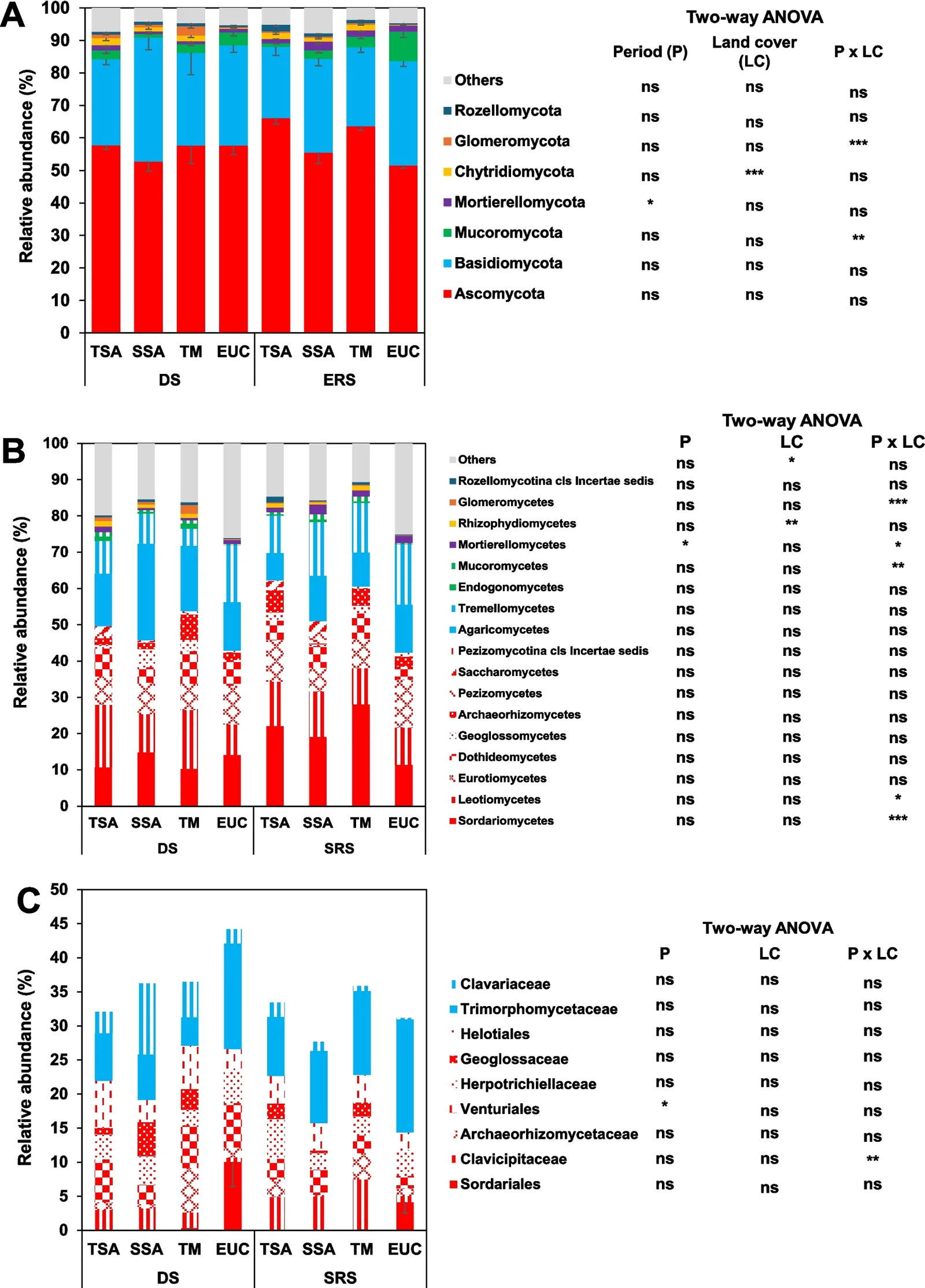
Comparisons of (**A**) phylum level, (**B**) class level (representative phyla with % ASV > 1), and (**C**) family level (representative families with %ASV > 5) of relative abundances of soil microbial ITS gene obtained from four land use types. TSA: Tree fallow, SSA: Shrub fallow, TM: Degraded land, EUC: Eucalyptus forest plantation, DS: dry season, SRS: start of rainy season (December). Data are means relative abundances − SE (*n* = 4). ***, **, and * indicate significance at *p* < 0.001, 0.01, and 0.05, respectively, ns: indicates not significant. *p*-values are based on the results of two-way ANOVA

*Ascomycota* dominated the fungal phyla, accounting for 52.69% to 66.10% of the total abundance (Fig. 4A), followed by *Basidiomycota* (21.93% to 38.22%). Neither P nor LC affected the abundance of these phyla. *Mortierellomycota* increased in SRS, with a lower abundance in TSA. Chytridiomycota was more abundant in the TSA and SSA land covers and less abundant in the EUC land cover. *Mucoromycota* varied by period: lower in SSA and higher in EUC during DS but lower in TSA and higher in EUC in SRS. *Glomeromycota* had a relatively high abundance in TM during DS. At the class level, *Sordariomycetes* (*Ascomycota*) had a greater abundance in SRS than in DS, except in EUC (Fig. 4B). *Mortierellomycetes* were more abundant in SRS than in DS, and *Rhizophydiomycetes* were less abundant in EUC. For fungal families (Fig. 4C), no interaction between P and LC was detected, and no effects of P and LC were detected. *Sordariales* were mainly found in EUC, whereas *Venturiales* were more abundant in DS than in SRS.

### Soil Parameters Driving the Soil Microbial Community Composition

Pearson’s correlation coefficient was used to assess the relationships between microbial abundance, functional genes, and soil properties. Soil pH positively influenced most of the targeted genes (Table 4). *ITS* abundance was correlated with soil P_tot_, whereas *16S rRNA* abundance was linked to P_Ols_. P_Ols_ was positively correlated with the *phoD* and betaine genes. Soil texture influenced microbial gene abundance, with clay negatively correlated with the betaine, *gcd*, and *nifD* genes but positively correlated with betaine and *phoD*, and silt negatively correlated with these genes (Table 4).

**Table 4:** Pearson’s correlation coefficients of soil microbial relative abundance and functional genes with soil properties

	pH	P_Ols_	P_tot_	N_tot_	C_org_	C/N	Clay	Sand	Silt
*16S rRNA*	0.56***	0.43**	0.27	−0.09	−0.04	0.07	−0.11	0.12	−0.07
*ITS*	0.36*	0.18	0.35*	−0.16	0.03	0.26	0.01	0	−0.03
*Betaine*	0.32*	0.62***	−0.06	−0.05	−0.12	−0.09	−0.40**	0.48***	−0.37**
*phoD*	0.31*	0.67***	0.13	−0.05	−0.12	−0.11	−0.19	0.28*	−0.33*
*gcd*	0.39**	0.27	0.09	0.05	0.13	0.09	−0.36*	0.25	0.15
*nifD*	0.32*	0.19	0.08	−0.09	0.05	0.17	−0.30*	0.24	0.05

P_ols_: Olsen P, P_tot_: total phosphorus, C_org_, organic carbon, N_tot_: total nitrogen

Significance level: * < 0.05, ** < 0.01, *** < 0.001

Pearson’s correlation analysis (Supplementary Fig. S5) was used to examine bacterial and fungal abundance in relation to soil pH and P_Ols_ across seasons. Both abundances correlated significantly with soil pH (Supplementary Fig. S5 A, S5 C), with bacteria showing a stronger correlation (*p* < 0.001) than fungi (*p* < 0.05). The microbial abundances were highest in SRS, with pH values ranging from 5.0 to 5.8, but lower in DS, with a more acidic pH. Available P was significantly correlated with bacterial abundance (*p* < 0.01) (Supplementary Fig. S5B) but not with fungal abundance and was generally greater in SRS, reflecting increased bacterial abundance with P_Ols_.

**Fig. 5:**
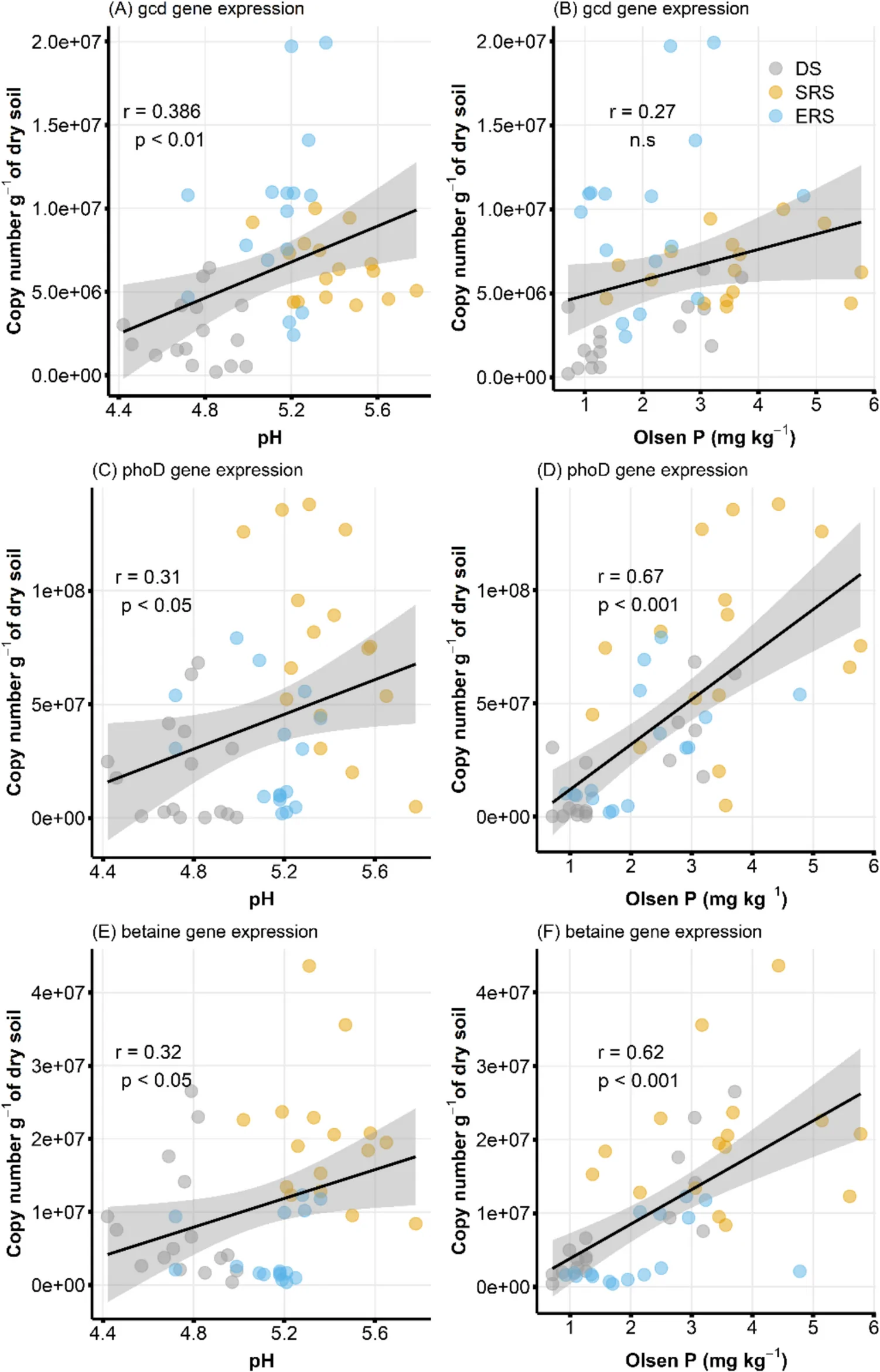
Correlation between P-related gene expression with pH and Olsen P (Pearson’s coefficient). DS: Dry season, SRS: start of rainy season, ERS: end of the rainy season

Figure 5 showed the correlations of functional genes with soil pH and P_Ols_, considering seasonal variations. The soil pH was significantly correlated with *gcd* (*p* < 0.01) (Fig. 5A), *phoD* (*p* < 0.05) (Fig. 5C), and betaine (*p* < 0.05) (Fig. 5E) gene abundance. Soils with pH values ranging from 5.0 to 5.8 presented relatively high *phoD* and betaine gene abundances, whereas the relatively low pH (< 5.0) in DS was correlated with relatively low *gcd* gene abundance. P_Ols_ was significantly correlated with the *phoD* (*p* < 0.01) (Fig. 5D) and betaine (*p* < 0.01) (Fig. 5F) genes, with higher abundances linked to higher _POL_ levels.

While the Pearson correlation highlighted relationships between microbial abundance and soil variables, it did not pinpoint the most explanatory factors. A multiple linear regression model indicated that soil pH and total/available P were key drivers of microbial abundance (Supplementary Table 7). P availability explained 34.6% and 46.54% of the *phoD* and betaine gene variation, respectively. The soil texture also played a role: clay influences P-solubilizing (*gcd*) and N_2_-fixing (*nifD*) bacteria, whereas silt influences betaine gene abundance. The effects of soil properties on microbial composition varied seasonally (Supplementary Table 8). In DS, P_Ols_ accounted for 45.30% of the *16S rRNA* and 24.99% of the *ITS* relative abundance, whereas different patterns emerged in SRS, indicating seasonal effects on microbial communities.

**Fig. 6:**
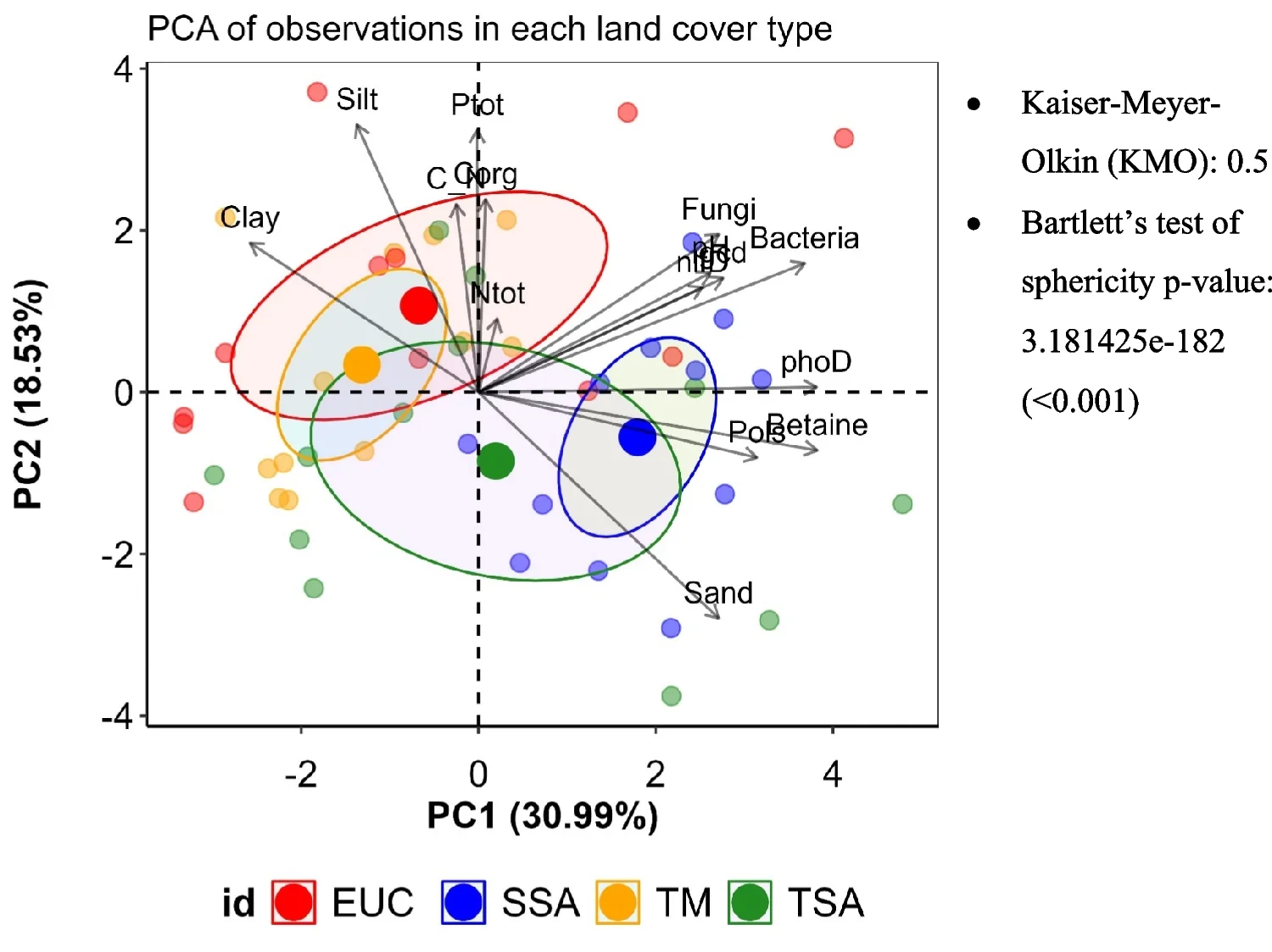
Principal component analysis between land cover types, soil properties, and microbial gene abundances. TSA: Tree fallow, SSA: Shrub fallow, TM: Degraded land, EUC: Eucalyptus forest plantation

PCA of the soil physicochemical and microbial parameters (Fig. 6) explained 49.52% of the variance. PC1 (30.99%) contrasted betaine and *phoD* gene abundance, microbial abundance, P_Ols_, and soil texture, linking high P availability and microbial gene abundance to SSA rather than TM. PC2 (18.53%) associated total P, SOC, the C/N ratio, and silt with TSA vs. EUC, where EUC presented higher values.

## Discussion

This study investigated how soil physicochemical properties, microbial diversity, and ecosystem functions interact in Madagascar’s tropical forests, which feature diverse land covers and marked seasonal variation. While microbial shifts associated with land cover changes have been well documented, the mechanisms by which soil properties influence these shifts remain less understood, particularly in tropical environments. Consistent with previous research, our findings highlight that both land cover types and seasonal changes significantly influence soil chemical properties [38–40]. The dry season (June) was characterized by lower moisture and cooler temperatures, whereas the rainy season (December and March) was characterized by warmer and wetter conditions. These environmental changes influenced microbial activity, nutrient cycling, and the decomposition of organic matter, contributing to temporal variability in both soil chemistry and microbial communities. Bacterial communities showed strong responses to seasonal moisture, corroborating earlier reports on their sensitivity to water availability [41, 42]. For example, *Actinobacteria* were more abundant during the rainy season, underscoring their known capacity for rapid recovery following dry spells and their role in carbon cycling through organic material breakdown. *Mycobacteriaceae*, a notable member of this phylum, exemplifies this resilience. Similarly, fungal communities responded to seasonal conditions, with *Mortierellomycota* showing increased relative abundance during the rainy season, especially in shrub fallow systems. This trend supports the findings that fungi are favored under wetter conditions, which enhances carbon turnover and nutrient cycling [43]. While eucalyptus plantations exhibited higher fungal abundance, likely due to ectomycorrhizal associations [13], their overall diversity was lower than that in tree and shrub fallows. This likely reflects the lower structural and species complexity in monocultures, which provide fewer ecological niches. Seasonal increases in phosphorus-cycling genes (e.g., *phoD*, *gcd*, betaine) in tree and shrub fallows suggest that moisture, possibly combined with reduced acidity, enhances microbial phosphorus mobilization during the rainy season.

Numerous studies have linked microbial structure and function to environmental variables such as soil texture [44], land use [45, 46], pH [46, 47], and nutrients content [45, 48]. Among these, soil pH emerged as a consistently strong predictor of microbial community composition across land covers and seasons [47, 49]. *Acidobacteria*, *Actinobacteria*, *Bacteroidetes*, and *Proteobacteria* were more strongly associated with pH gradients than with geographic location, a finding that aligns with their known pH sensitivities. While fungi tend to be functionally stable across pH ranges, bacterial communities exhibit narrower tolerance ranges, rendering them more sensitive to pH fluctuations. A pH range of 6.0 to 7.0 is generally considered optimal, as it promotes the solubility and uptake of essential nutrients, thereby supporting soil fertility and plant productivity [49].

Our data show that pH varied significantly across land covers and seasons, with more acidic conditions in tree and shrub fallows, and eucalyptus plantations during the dry season. These shifts in soil pH, combined with variations in moisture and texture, influenced both microbial richness and density and the abundance of functional genes involved in nutrient cycling. This highlights the complex interplay between soil pH, nutrient availability, vegetation type, and seasonal variation in shaping microbial communities in tropical soils. To better understand the relationship between soil chemistry and microbial properties, we conducted regression analyses. These revealed that soil organic carbon and total nitrogen were strong predictors of fungal abundance, while available phosphorus was more closely associated with bacterial abundance—particularly in tree and shrub fallows where its levels were elevated. Microbial populations in degraded lands remained low despite high organic carbon and nitrogen contents, suggesting that these nutrients may be less bioavailable or effective in promoting microbial activity under such conditions. This emphasizes that nutrient availability, rather than total nutrient content, is more critical for supporting microbial functional and taxonomic diversity. The functional contributions of microbial communities also appeared to vary by land cover. Shrub fallows, rich in nitrogen-fixing bacteria, likely enhance nitrogen availability, whereas eucalyptus systems may experience nutrient leaching, reducing overall soil fertility. The higher microbial richness and diversity observed in the tree and shrub fallows likely reflect the greater vegetation heterogeneity, carbon inputs from root exudates, finer root systems, and litter quality—all of which support microbial growth and community structure [50]. In contrast, eucalyptus plantations and degraded land showed lower microbial diversity, possibly due to limited input and greater disturbances.

Although the soil moisture was not directly measured, inferred relationships suggest that porous soils in shrub fallows may retain more water and support better oxygen exchange, creating conditions that favor microbial proliferation [51, 52]. Conversely, the clay-rich soils of eucalyptus plantations may impede drainage and reduce microbial activity. These findings align with studies emphasizing the importance of plant species composition in shaping soil microbial structure. For instance, Zhang et al. [53] reported that mix-cropping systems support higher microbial biomass and activity than mono-specific systems, likely due to more diverse organic inputs and wider range of ecological niches.

While root exudates were not directly analyzed in this study, their importance in shaping microbial structure is well-established. Seasonal microbial shifts also varied at finer taxonomic levels. Although *Proteobacteria* did not show seasonal differences at the phylum level, specific groups such as *Gammaproteobacteria* increased in eucalyptus sites, potentially in response to organic compound degradation [54]. Meanwhile, nitrogen-fixing taxa like *Bradyrhizobiaceae* (*α-Proteobacteria*) were more abundant in shrub and tree fallows, highlighting the role of moisture in stimulating functionally important microbes. *Acidobacteria* were particularly abundant in eucalyptus plantations, consistent with previous reports on their affinity for low pH and the acidifying effect of eucalyptus vegetation [55, 56]. Degraded systems exhibited notably lower microbial abundance, which may slow decomposition and carbon turnover despite similar climatic conditions. Collectively, these findings underscore the critical influence of vegetation structure and composition—beyond soil texture—on microbial diversity and function. Land cover changes that alter plant diversity and soil chemistry can therefore trigger cascading effects on microbial ecology and biogeochemical cycling.

## Conclusions

This study explored the dynamics of soil microbial communities and chemical properties across different land covers and seasons in Madagascar’s tropical forests. Our findings suggest that microbial communities are shaped by complex interactions between soil pH, phosphorus availability, and seasonal moisture fluctuations. Furthermore, this study underscores the importance of native vegetation in maintaining microbial diversity, which support nutrient cycling processes crucial for soil health and ecosystem restoration. These insights can inform reforestation and land management efforts aimed at restoring soil function and resilience in tropical regions. By addressing the mechanistic links between soil properties and microbial function, our study contributes to filling a key knowledge gap in tropical microbial ecology. However, while strong correlations between microbial composition and soil parameters were identified, causality remains unresolved. Experimental manipulations of soil conditions and microbial assemblages will be essential for disentangling these relationships and to guide sustainable land management practices in tropical landscapes.

## Supplementary Information

Below is the link to the electronic supplementary material.

Supplementary file1 (DOCX 1304 kb)

Supplementary file2 (DOCX 90 kb)

## Data Availability

The raw sequences have also been deposited in the DDBJ Sequence Read Archive database under the accession numbers DRR627433–DRR627464 for the 16S rRNA sequences and DRR627465–DRR627496 for the ITS sequences. The other data are provided within the manuscript or supplementary information files.
