# Supplementary material for: Land Cover and Seasonal Variations Shape Soil Microbial Communities and Nutrient Cycling in Madagascar Tropical Forests
Source: Microb Ecol. 2025 Jun 4;88:60. doi: 10.1007/s00248-025-02561-w (PMC12137386; doi:10.1007/s00248-025-02561-w)
Supplement: Supplementary file 1 [file 248_2025_2561_MOESM1_ESM.docx]

A

B

**Madagascar**

Supplementary Fig. S1. Geographical position of sampling sites (A) and the composition of plant species in the sites (B)

TSA: Tree fallow, SSA: Shrub fallow, TM: Degraded land, EUC: Eucalyptus forest plantation

Supplementary Fig. S2. Soil texture triangle by land cover types

Supplementary Fig. S3. Nonmetric multidimensional scaling (NMDS) ordination of bacterial beta-diversity through the season

TSA: Tree fallow, SSA: Shrub fallow, TM: Degraded land, EUC: Eucalyptus forest plantation, DS: Dry season, ERS: End of the rainy season

Supplementary Fig. S4. Nonmetric multidimensional scaling (NMDS) ordination of fungal beta-diversity through the season

TSA: Tree fallow, SSA: Shrub fallow, TM: Degraded land, EUC: Eucalyptus forest plantation; DS: Dry season, ERS: End of the rainy season

Supplementary Fig. S5. Correlation between soil total bacteria and total fungi relative abundance with pH and P_Ols_ (Peason's coefficient).

DS: Dry season, SRS: Start of rainy season, ERS: End of the rainy season

Supplementary Fig. S6: Soil moisture content during DNA extraction.

TSA: Tree fallow, SSA: Shrub fallow, TM: Degraded land, EUC: Eucalyptus forest.
