# Supplementary material for: Land Cover and Seasonal Variations Shape Soil Microbial Communities and Nutrient Cycling in Madagascar Tropical Forests
Source: Microb Ecol. 2025 Jun 4;88(1):60. doi: 10.1007/s00248-025-02561-w (PMC12137386; doi:10.1007/s00248-025-02561-w)
Supplement: Supplementary file 2 — Supplementary file2 (DOCX 90 kb) [file 248_2025_2561_MOESM2_ESM.docx]

Supplementary Table S1. Empirical meteorological data during the study period from local weather stations

| Year | Month | Rainfall | | RH | Tmin | Tmax |
| --- | --- | --- | --- | --- | --- | --- |
|  |  | mm/day | mm/month | % | ºC | |
| 2022 | May | 5.76 | 178.44 | 91.48 | 14.31 | 21.18 |
|  | **June** | **5.42** | **162.59** | **92.94** | **12.37** | **18.11** |
|  | July | 6.12 | 189.72 | 93.27 | 11.95 | 17.22 |
|  | August | 2.45 | 75.86 | 89.7 | 11.54 | 19.09 |
|  | September | 2.21 | 66.41 | 87.73 | 11.77 | 19.54 |
|  | October | 1.18 | 36.62 | 84.62 | 13.08 | 22.59 |
|  | November | 5.36 | 160.9 | 85.26 | 15.73 | 24.71 |
|  | **December** | **10.47** | **324.54** | **88.27** | **16.87** | **24.72** |
| 2023 | January | 15.62 | 484.35 | 89.15 | 17.77 | 24.68 |
|  | February | 14.58 | 408.13 | 90.06 | 17.42 | 24.46 |
|  | **March** | **15.4** | **477.49** | **90.98** | **17.56** | **24.62** |
|  | April | 8.46 | 253.67 | 90.96 | 16.45 | 23.35 |
|  | May | 8.3 | 237.19 | 90.91 | 15.06 | 22.39 |

mm: millimeter, Tmin: minimal temperature, Tmax: maximal temperature. Values in bold are those from the sampling months. Data is obtained from the local weather station in Moramanga district.

Supplementary Table S2. Land cover dominant species according to Styger et al.2007

| **Land cover** | **Dominant species** |
| --- | --- |
| Tree fallow (TSA) | -*Trema orientalis* |
|  | *-Harungana madagascariensis* |
| Shrub fallow (SSA) | *-Psiadia altissima* |
|  | *-Rubus moluccanus* |
|  | *-Lantana camara* |
| Degraded land (TM) | *-Imperata cylindrica* |
|  | *-Aristida sp.* |
| Eucalyptus forest (EUC) | *-Eucalyptus robusta* |

Supplementary Table S3. Amplification efficiencies and r^2^ values during microbial genes abundance determination by qPCR

| Microbial genes | Amplification efficiency ranges (%) | r^2^ values |
| --- | --- | --- |
| 16S rRNA (bacteria) | [101.6] | [0.998] |
| ITS (fungi) | [79.9] | [0.998] |
| *gcd* | [80.7] | [0.999] |
| *phoD* | [85.0] | [0.999] |
| *nifD* | [72.7] | [0.999] |
| *Betaine* | [87.0] | [0.996] |

Supplementary Table S4. Reads and ASVs of 16S rRNA and ITS amplicons

| Land cover | Rep | Sampling period |  | 16S rRNA reads | | 16S rRNA | ITS | |
| --- | --- | --- | --- | --- | --- | --- | --- | --- |
|  |  |  |  | Raw | Valid | ASVs | Reads | ASVs |
|  |  |  | **Max** | **16472** | **16427** | **810** | **44166** | **428** |
|  |  |  | **Min** | **13375** | **13339** | **531** | **42591** | **240** |
| TSA | 1 | DS |  | 14585 | 14477 | 594 | 42653 | 356 |
| TSA | 2 | DS |  | 15459 | 15443 | 718 | 42711 | 387 |
| TSA | 3 | DS |  | 15138 | 15105 | 684 | 43409 | 303 |
| TSA | 4 | DS |  | 14604 | 14488 | 695 | 43325 | 360 |
| SSA | 1 | DS |  | 14331 | 14291 | 626 | 43800 | 417 |
| SSA | 2 | DS |  | 13961 | 13957 | 779 | 43280 | 417 |
| SSA | 3 | DS |  | 13797 | 13772 | 595 | 42838 | 311 |
| SSA | 4 | DS |  | 13554 | 13476 | 605 | 43068 | 368 |
| TM | 1 | DS |  | 15964 | 15929 | 800 | 43382 | 377 |
| TM | 2 | DS |  | 15926 | 15864 | 767 | 43369 | 357 |
| TM | 3 | DS |  | 16385 | 16308 | 810 | 43313 | 349 |
| TM | 4 | DS |  | 16472 | 16427 | 711 | 43126 | 291 |
| EUC | 1 | DS |  | 15620 | 15620 | 627 | 43892 | 294 |
| EUC | 2 | DS |  | 15217 | 15206 | 643 | 43727 | 240 |
| EUC | 3 | DS |  | 15626 | 15610 | 618 | 42957 | 281 |
| EUC | 4 | DS |  | 15495 | 15483 | 600 | 43479 | 288 |
| TSA | 1 | ERS |  | 13796 | 13780 | 627 | 42953 | 421 |
| TSA | 2 | ERS |  | 13639 | 13610 | 698 | 43560 | 428 |
| TSA | 3 | ERS |  | 14241 | 14222 | 726 | 44166 | 401 |
| TSA | 4 | ERS |  | 14572 | 14509 | 781 | 43144 | 386 |
| SSA | 1 | ERS |  | 14517 | 14504 | 544 | 43422 | 344 |
| SSA | 2 | ERS |  | 14693 | 14628 | 622 | 43150 | 333 |
| SSA | 3 | ERS |  | 14141 | 14110 | 554 | 43425 | 289 |
| SSA | 4 | ERS |  | 13375 | 13339 | 610 | 42786 | 377 |
| TM | 1 | ERS |  | 14577 | 14558 | 675 | 43111 | 247 |
| TM | 2 | ERS |  | 14973 | 14939 | 716 | 43071 | 330 |
| TM | 3 | ERS |  | 14749 | 14711 | 688 | 43709 | 337 |
| TM | 4 | ERS |  | 14708 | 14698 | 648 | 43833 | 252 |
| EUC | 1 | ERS |  | 14898 | 14865 | 531 | 42591 | 251 |
| EUC | 2 | ERS |  | 14511 | 14496 | 595 | 43295 | 388 |
| EUC | 3 | ERS |  | 14145 | 14109 | 553 | 43184 | 244 |
| EUC | 4 | ERS |  | 13598 | 13591 | 537 | 43158 | 258 |

TSA: Tree fallow, SSA: Shrub fallow, TM: Degraded land, EUC: Eucalyptus forest plantation, DS: dry season, ERS: early rainy season, ASV: Amplicon Sequence Variant

Supplementary Table S5. Soil bacterial diversity indices of the different land covers

| Period | Land cover | Shannon index | Simpson index |
| --- | --- | --- | --- |
| DS | TSA | 5.825 ± 0.078 cd | 0.9938 ± 0.00067 cd |
|  | SSA | 5.858 ± 0.094 de | 0.9942 ± 0.00075 cd |
|  | TM | 5.974 ± 0.048 e | 0.9940 ± 0.00050 bc |
|  | EUC | 5.721 ± 0.016 bc | 0.9929 ± 0.00011 d |
| SRS | TSA | 5.975 ± 0.055 e | 0.9952 ± 0.00027 d |
|  | SSA | 5.615 ± 0.051 ab | 0.9912 ± 0.00057 b |
|  | TM | 5.800 ± 0.047 cd | 0.9915 ± 0.00088 b |
|  | EUC | 5.567 ± 0.041 a | 0.9897 ± 0.00080 a |
| Period (P) | | * | *** |
| Land cover (LC) | | *** | *** |
| P x LC | | * | ** |

TSA: Tree fallow, SSA: Shrub fallow, TM: Degraded land, EUC: Eucalyptus forest plantation, DS: dry season, SRS: start of rainy season. Data are means ± SE (n = 4). ***, **, and * indicate significance at *p* < 0.001, 0.01, and 0.05, respectively. p-values are based on the results of two-way ANOVA and means are compared using the T HSD comparison method. Means followed by the same letters for each variable are not significantly different.

Supplementary Table S6. Soil fungal diversity indices of the different land covers

| Land cover | Shannon index | Simpson index |
| --- | --- | --- |
| TSA | 4.917 ± 0.052 c | 0.9821 ± 0.001 c |
| SSA | 4.729 ± 0.060 b | 0.9739 ± 0.003 b |
| TM | 4.643 ± 0.085 b | 0.9754 ± 0.004 b |
| EUC | 4.081 ± 0.115 a | 0.9477 ± 0.006 a |
| Period (P) | ns | ns |
| Land cover (LC) | *** | *** |
| P x LC | ns | ns |

TSA: Tree fallow, SSA: Shrub fallow, TM: Degraded land, EUC: Eucalyptus forest plantation, DS: dry season, ERS: early rainy season. Data are means ± SE (n = 8 since P x CL was not significant). *** indicates significance at *p* < 0.001. p-values are based on the results of two-way ANOVA and means are compared using the T HSD comparison method. Means followed by the same letters for each variable are not significantly different.

Supplementary Table S7. Relative importance of soil physicochemical properties on soil microbial relative abundance (multiple linear regression)

|  | pH | P_Ols_ | P_tot_ | C_org_ | N_tot_ | C/N | Clay | Silt | Sand | POV (%) |
| --- | --- | --- | --- | --- | --- | --- | --- | --- | --- | --- |
| All data | | | | | | | | | | |
| *16S rRNA* | 49.2 | 20.6 | 16.9 | - | 9.3 | - | - | 4.08 | - | 62.14 |
| *ITS* | 30.8 | - | 36.7 | - | 10.5 | 12.3 | - | 9.72 | - | 49.11 |
| *gcd* | - | 16.2 | 11.5 | - | 5.21 | - | 30.8 | 15.8 | 20.6 | 33.82 |
| *phoD* | 8.54 | 34.6 | 4.46 | 2.96 | - | 1.26 | - | 8.46 | - | 60.25 |
| *Betaine* | 12 | 46.5 | 2.61 | 5.31 | - | 2.52 | 15.2 | 15.8 | - | 55.06 |
| *nifD* | 29.9 | - | 15.8 | - | 15.3 | - | 39 | - | - | 25.81 |

P_Ols_: Olsen P, P_tot_: total phosphorus, C_org_, N_tot_: total nitrogen, POV: Proportion of variance

Supplementary Table 8: Relative importance of soil physicochemical properties on soil microbial relative abundance for each sampling season (multiple linear regression)

| **Genes** | **pH** | **P_Ols_** | **P_tot_** | **C_org_** | **N_tot_** | **C/N** | **Clay** | **Silt** | **Sand** | **POV (%)** |
| --- | --- | --- | --- | --- | --- | --- | --- | --- | --- | --- |
| DS (June 2022) | | | | | | | | | | |
| *16S rRNA* | 19.63 | 45.3 | - | - | - | 9.59 | 8.98 | 5.67 | 10.84 | 78.95 |
| *ITS* | 6.67 | 24.99 | 9.49 | 18.37 | 20.98 | 13.43 | - | 6.07 | - | 82.02 |
| *gcd* | 13.82 | 56.39 | - | - | - | - | - | - | 29.79 | 77.86 |
| *phoD* | 9.28 | - | 14.47 | - | - | 11.73 | 64.53 | - | - | 67.19 |
| *Betaine* | 6.8 | 79.68 | 2.98 | 3.34 | 3.59 | 3.61 | - | - | - | 93.05 |
| *nifD* | 39.96 | - | - | - | - | 4.1 | 39.56 | 16.38 | - | 80.45 |
| SRS (December 2022) | | | | | | | | | | |
| *16S rRNA* | - | - | 33.44 | - | 56.55 | 10.01 | - | - | - | 74.18 |
| *ITS* | 7.26 | 19.17 | 23.85 | - | - | 4.13 | 18.62 | 12.11 | 14.85 | 70.76 |
| *gcd* | - | - | 17.61 | - | - | - | 23.15 | 34.57 | 24.67 | 57.84 |
| *phoD* | 42.67 | - | - | - | - | - | - | 57.33 | - | 54.24 |
| *Betaine* | - | - | 6.34 | - | - | - | 20.47 | 46.82 | 26.37 | 56.89 |
| *nifD* | - | 17.48 | 39.47 | - | 16.36 | 26.68 | - | - | - | 72.3 |
| ERS (March 2023) | | | | | | | | | | |
| *16S rRNA* | 6.22 | 24.23 | - |  | 28.46 | 9.65 | 16.95 | - | 14.49 | 79.98 |
| *ITS* | - | 16.62 | - | 51.68 | - | 31.7 | - | - | - | 37.1 |
| *gcd* | - | 24.32 | - | 5.54 | - | 4.2 | 56.99 | 8.94 | - | 78.69 |
| *phoD* | - | 78.06 | - | 21.94 | - | - |  | - | - | 46.14 |
| *Betaine* | - | 31.95 | - | 11.94 | - | - | 56.11 | - | - | 72.53 |
| *nifD* | - | 9.62 | - | 15.25 | - | 3.33 | 71.8 | - | - | 70.71 |

DS: Dry season, SRS: Starting rainy season, ERS: End of rainy season
